# Conformational restriction of a type II FMS inhibitor leading to discovery of 5-methyl-*N*-(2-aryl-1*H*-benzo[d]imidazo-5-yl)isoxazole-4-carboxamide analogues as selective FLT3 inhibitors

**DOI:** 10.1080/14756366.2019.1671837

**Published:** 2019-10-01

**Authors:** Daseul Im, Hyungwoo Moon, Jingwoong Kim, Youri Oh, Miyoung Jang, Jung-Mi Hah

**Affiliations:** College of Pharmacy and Institute of Pharmaceutical Science and Technology, Hanyang University, Ansan, Korea

**Keywords:** FMS, benzimidazole, conformational restriction, FLT3

## Abstract

A series of 4-arylamido 5-methylisoxazole derivatives incorporating benzimidazole was designed and synthesised by conformational restriction of an in-house type II FMS inhibitor. Kinase profiling of one compound revealed interesting features, with increased inhibitory potency towards FLT3 and concomitant loss of potency towards FMS. Several benzimidazole derivatives **5a–5g** and **6a–6c** containing various hydrophobic moieties were synthesised, and their inhibitory activity against FLT3 was evaluated. Specifically, **5a**, 5-methyl-*N*-(2-(3-(4-methylpiperazin-1-yl)-5-(trifluoromethyl)phenyl)-1*H*-benzo[d]imidazole-5-yl) isoxazole-4-carboxamide, exhibited the most potent inhibitory activity against FLT3 (IC_50 _= 495 nM), with excellent selectivity profiles.

## 1. Introduction

Tyrosine kinases facilitate the majority of intracellular signal transduction by catalysing the transfer of the γ-phosphoryl group of ATP^1^. The first epidermal growth factor receptor (EGFR) kinase inhibitors were reported in the 1980s. Since then, improved understanding of binding modes and ligand interactions has led to development of numerous kinase inhibitors with various structure and inhibition profiles^2^.

**Figure 1.**
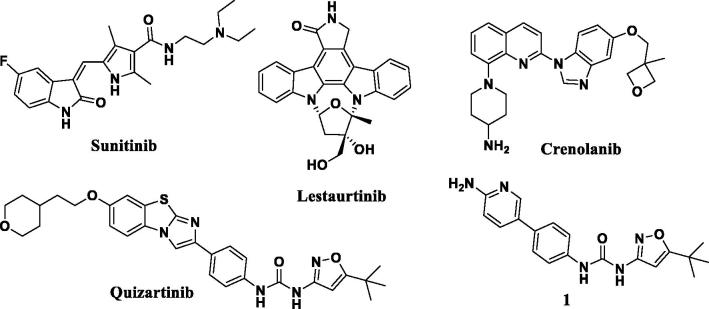
Chemical structures of known FLT3 inhibitors and compound 1, inhibitor of FMS, FLT3, and Kit (**1**).

The list of known kinase targets is vast and includes the receptor tyrosine kinase FMS-like tyrosine kinase 3 (FLT3). Importantly, FLT3 mediates the survival, proliferation, and differentiation of haematopoietic stem and progenitor cells in the majority of patients with acute myelogenous leukaemia (AML)^3–6^. Various inhibitors of FLT3 have been developed, some of which have advanced to clinical trials with the goal of improving clinical outcomes specifically for patients with AML associated with FLT3 mutations (Figure 1). Several early FLT3 inhibitors including sunitinib, midostaurin, and lestaurtinib demonstrated significant promise in preclinical models of FLT3 mutant AML^7^. Unfortunately, many of these compounds failed to achieve stable FLT3 inhibition in early clinical trials, resulting in only transient decreases in peripheral blast counts. These results prompted the development of second-generation FLT3 inhibitors, epitomised by the novel agent quizartinib^8^^,^^9^. We previously identified an interesting structural resemblance between quizartinib and a biaryl FMS inhibitor herein termed compound 1. In addition to FMS inhibition, compound 1 exhibits an IC_50_ of 1 nM against FLT-3 and KIT in competitive-binding assays performed *in vitro*^10^.

Conformational rigidification^11^ is a useful strategy in drug design to minimise entropy loss associated with ligands that adopt a preferred conformation for binding, improve isoform selectivity, and reduce the potential for drug metabolism. We previously employed this strategy to several type II FMS inhibitors^12^ to identify FLT3 inhibitors based on the structural similarity of these two kinases.

**Figure 2.**
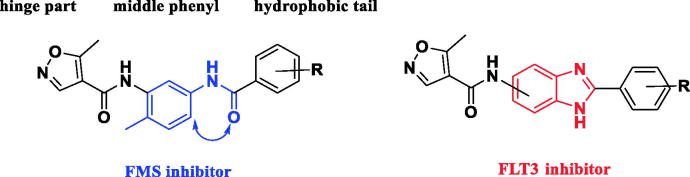
Design of benzimidazole derivatives as bioisosteres of the middle phenyl ring-amide-secondary hydrophobic aromatic ring.

Type II FMS inhibitors consist of three parts, a hydrogen-bonding hinge, a central phenyl ring, and a secondary hydrophobic aromatic ring that facilitates binding to the DFG pocket^13^. Amide or urea linkages connect the middle phenyl ring and secondary hydrophobic aromatic ring. In the present study, we utilised conformational restriction of the connection to synthesise a novel heterocyclic scaffold (Figure 2). Specifically, we utilised a benzimidazole group as a rigid substitute for the middle phenyl ring-amide-secondary hydrophobic aromatic ring. Benzimidazole is a well-known privileged structure in medicinal chemistry that exhibits diverse biological activities^14^. Through our introduction of this structure into our in-house type II kinase inhibitor, we identified several novel FLT3 inhibitors with improved selectivity.

## 2. Results and discussion

**Scheme 1.**
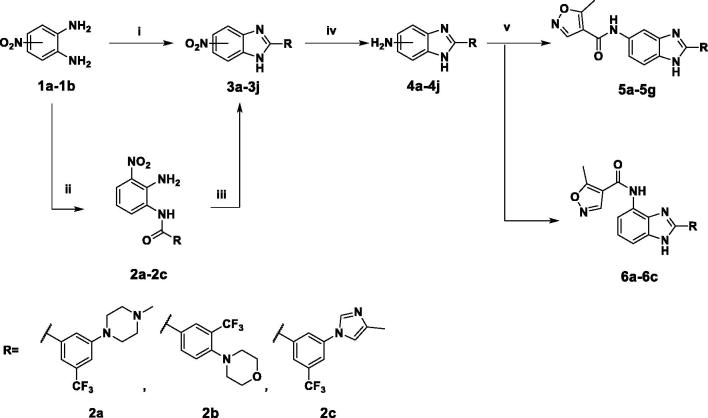
Synthesis of 1H-benzimidazolyl isoxazole-4-carboxamide derivatives. (i) benzoic acid, POCl_3_, µW, 192 °C, 10 min for **3a–3g**; (ii) benzoyl chloride, MC/CAN = 2:1, rt, 2 h; (iii) HCl/H_2_O/AcOH, 150 °C, 30 min for **3h–3j**; (iv) H_2_ , Pd/C, MeOH 1 h for **4a**, **4b**, **4c**, **4g**, **4h**, **4i** or SnCl_2_, EtOH, 90 °C, 1 h for **4d**, **4e**, **4f** or Fe, AcOH/H_2_O/EtOH, 60 °C for **4j**; (v) 5-methylisoxazole-4-carbonyl chloride, THF, 65 °C, 1 h

The general synthesis of 3-carbonyl-1*H*-benzimidazolyl isoxazole-4-carboxamide (**5a–g, 6a–c**) is shown in Scheme 1 (See Supplementary Material). A solution of 4-nitro-1,2-phenylenediamine (**1a**) and substituted benzoic acid or pyrazole carboxylic acid in phosphorus oxychloride was reacted under microwave irradiation at 192 °C for 10 min to give the core intermediate benzimidazoles (**3a-g**)^15^. For 1,2-diamino-3-nitrobenzene (**1b**), the core structure was synthesised in two sequential steps. First, benzamide (**2a-c**) formation was achieved using triethylamine and benzoyl chloride in a mixture of CH_2_Cl_2_/acetonitrile (2:1), which was reacted in a solution of concentrated aqueous HCl (35%) and acetic acid under microwave irradiation at 150 °C to give the core intermediates **3h–j**^16^. The nitro group of benzimidazole was then reduced to amines **4a–j** and coupled with isoxazole chloride to produce carboxamides (**5a–g, 6a–c**).

All the benzimidazole compounds **5a–5g**, **6a–6c** were evaluated for activity against FLT3 kinase, the results of which are shown in Table 1. The synthesised compounds exhibited selective activity against FLT3, especially those that incorporated piperazine, morpholine, or imidazole moieties in the hydrophobic tail. Amongst the compounds evaluated, **5a** showed the most potent activity against FLT3, with an IC_50_ value of 495 nM.

**Table 1.** Enzymatic activities of 1*H*-benzimidazolyl isoxazole-4-carboxamide derivatives.


		IC_50_ (µM)				IC_50_ (µM)	
No	R	FLT3	FMS	No	R	FLT3	FMS
5a		0.495	NA	**5f**		13.4	NA
5b		7.94	NA	**5g**		NA	NA
5c		∼10	NA	**6a**		2.25	NA
5d		2.33	NA	**6b**				13.9	NA
5e		1.62	9.98	**6c**		6.20	NA		
Staurosporine						1.13 nM	0.88 nM		

Structure activity relationships (SARs) were inferred from potency data. We first noted that the benzimidazole compounds exhibited different tendencies depending on the position of the substituent **R** group on the phenyl ring. With respect to the benzimidazole derivatives, the compounds that were 1,3,5-substituted (**5b**) or 1,3,4-substituted with benzoic acid (**5c**) retained their activity against FLT3, with IC_50_ values of 7.94 and ∼10 µM, respectively. Only the benzimidazole compound with a pyrazole moiety (**5g**) lost activity against FLT3.

**Figure 3.**
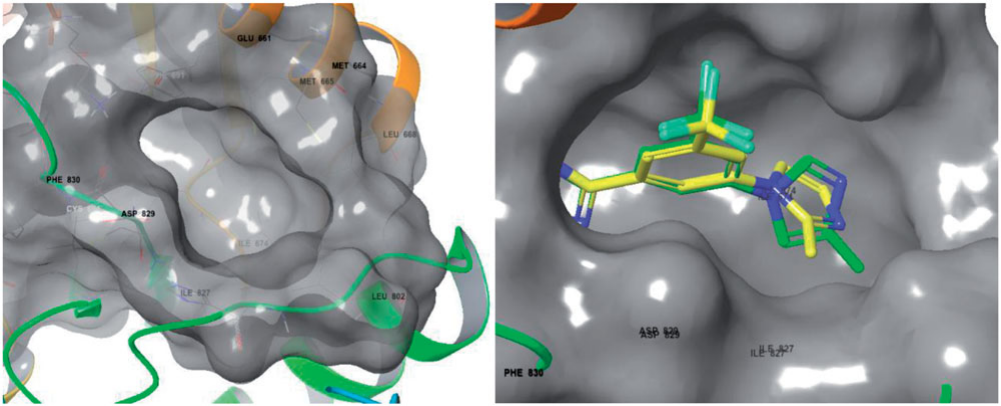
(Left) Solid surface view of the hydrophobic pocket formed by residues Ile 674, Leu 688, Met 799, Leu 802, and Ile 827 (PDB: 4RT7). (Right) Overlapped binding mode between **5d** (green) and **5f** (yellow) superimposed on a solid surface view of the hydrophobic pocket. (PDB : 4RT7)^17^.

The position of the methyl substituent on the imidazole ring was found to play an important role in inhibitory activity. Specifically, imidazole compounds incorporating a 4-substituted or 5-substituted methyl group (**5d–5e**) were 6–8 times more potent than the imidazole compound substituted at the 2-position (**5f**). There is a hydrophobic pocket surrounded by several hydrophobic residues (Ile 674, Leu 688, Met 799, Leu 802, Ile 827) adjacent to the ATP-binding site of FLT3 kinase. Docking studies showed that the methyl positions of **5d** and **5e** allowed these compounds to occupy this hydrophobic pocket more suitably than in **5f** (Figure 3). This observance may explain the differences in activity amongst the imidazole derivatives.

**Figure 4.**
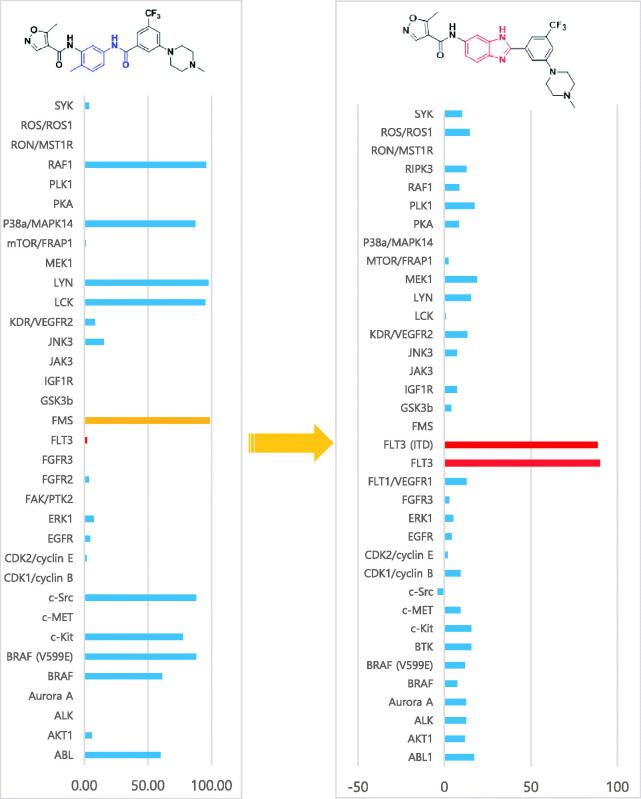
Kinase profiling according to chemical scaffold. The profiles of the FMS kinase inhibitor^13^ and benzimidazole derivative **5a** (10 μM) are shown.

We next investigated the effects of various substitutions of the benzimidazole ring. The 6-amide compounds (**5a**, **5c**, **5d**) were more potent, whilst 7-amide analogues (**6a**, **6b**, **6c**) exhibited slightly weak inhibitory activity against FLT3. **6a** was the most potent amongst the 7-amide derivatives. Comparison of the relative potencies of the 6-amide and 7-amide derivatives revealed similar tendencies (piperazine > imidazole > morpholine). We next performed a kinase panel screen for **5a** against more than 35 different kinases at a single-dose of 10 µM (Figure 4, screen performed in duplicate). **5a** achieved an excellent selectivity profile with an acquired inhibitory activity of 89.97% against FLT3 but without any significant activity against other protein kinases, especially FMS. Overall, this result indicates that the kinase activity profile and selectivity of the original FMS inhibitor changed dramatically as a result of the conformational restriction strategy.

**Figure 5.**
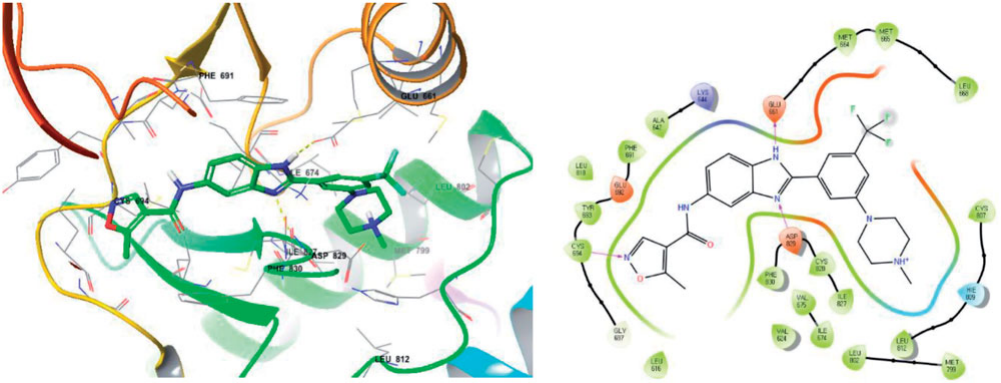
Docking structures between FLT3 (PDB: 4RT7) and the newly designed 1H-benzimidazolyl isoxazole-4-carboxamide derivative **5a**.

To better understand the interactions between the newly synthesised compounds and FLT3, we performed molecular docking studies of compound **5a** in the ATP-binding pocket of FLT3 (PDB: 4RT7) using Glide (SCHRODINGER software package Version 14.2), the results of which are shown in Figure 5. In the binding model, compound **5a** was tightly bound to the ATP-binding site of FLT3 via several hydrogen bonds, π-π interactions, a π-cation interaction, and an ionic interaction.

The predicted binding mode of **5a** is also shown in Figure 5. In the model, the nitrogen of 5-methylisoxazole as a hinge binder forms a hydrogen bond with the amino hydrogen of Cys 694. The benzimidazole moiety of **5a** forms two hydrogen bonds, one between the N–H of benzimidazole and the oxygen of Glu 661 and another between the nitrogen of benzimidazole and N–H of the Asp 829 backbone. Identification of these hydrogen bonds suggests that the benzimidazole moiety experiences a hybrid binding mode between the middle phenyl ring and the amide or urea linkage as designed. In addition, the N-methyl piperazine tail exhibited a hydrophobic interaction within the hydrophobic pocket surrounded by several residues (Ile 674, Met 799, Leu 802, Ile 827).

## 3. Conclusions

We designed and synthesised a series of 4-arylamido 5-methylisoxazole derivatives containing benzimidazole based on a conformational restriction strategy. Various analogues were synthesised and tested for inhibitory activity against FLT3. Compound **5a** displayed the most potent inhibitory activity against FLT3, with an IC_50_ of 495 nM. Using the conformational restriction strategy, we successfully altered protein kinase inhibitory activity from FMS to FLT3. In addition, we performed a kinase panel screen using compound **5a** for 34 different kinases at a single-dose of 10 µM in duplicate (Table 2). The results of the screen showed that the newly synthesised FLT3 inhibitors had excellent selectivity profiles towards FLT3 kinase. Considering that FLT3 is significantly associated with AML, the unique chemical scaffold described in this study may be valuable for developing new molecules as potential therapeutic agents for this disease. Indeed, the above findings provide a theoretical basis for further structural optimisation of 4-arylamido 3-methyl isoxazole derivatives as FLT3 inhibitors. Amongst potential derivatives, compound **5a** represents a promising lead for new therapeutics targeting AML due to its strong kinase selectivity profile.

**Table 2.** Enzymatic inhibition exerted by **5a** (10 μM) against 34 selected protein kinases.

Kinase	% Inhibition	Staurosporine IC_50_ (nM)
ABL1	17.095	31.0
AKT1	11.91	1.98
ALK	12.61	2.35
Aurora A	12.49	0.502
BRAF	7.31	7.59^a^
BRAF (V599E)	11.905	7.93^a^
BTK	15.58	23.4
c-Kit	15.545	8.69
c-MET	9.18	97.7
c-Src	0.00	67.8
CDK1/cyclin B	9.31	3.46
CDK2/cyclin E	1.785	1.97
EGFR	4.095	3.37
ERK1	5.18	73.2
FGFR3	2.805	14.7^b^
FLT1/VEGFR1	12.72	15.5
FLT3	89.97	10.6
FLT3 (ITD)	88.48	0.97
FMS	0.00	1.54
GSK3b	3.975	2.90
IGF1R	7.135	25.2
JAK3	0	640.0^c^
JNK3	7.12	8.60
KDR/VEGFR2	13.215	4.27
LCK	0.625	1.02
LYN	15.21	23.6
MEK1	18.82	150.7
MTOR/FRAP1	2.355	12.3^d^
P38a/MAPK14	0	0.55
PKA	8.455	77.3
PLK1	17.38	11.0
RAF1	8.475	80.5
RIPK3	12.705	0.14
RON/MST1R	0	0.39

a Data of GW5074^18^.

b Data of SCH772984^19^^,^^20^.

c Data of JNKI VIII^21^^,^^22^.

d Data of SB202190^23^.

## Supplementary Material

Supplemental Material
