# Supplementary material for: Conformational restriction of a type II FMS inhibitor leading to discovery of 5-methyl-N-(2-aryl-1H-benzo[d]imidazo-5-yl)isoxazole-4-carboxamide analogues as selective FLT3 inhibitors
Source: J Enzyme Inhib Med Chem. 2019 Oct 1;34(1):1716–21. doi: 10.1080/14756366.2019.1671837 (PMC6781469; doi:10.1080/14756366.2019.1671837)
Supplement: Supplemental Material [file IENZ_A_1671837_SM7716.pdf]

Supplementary Material

1. Molecular Modelling

Compounds were docked into the FLT3 structure (PDB: 4RT7). Protein and ligand preparations were performed with Schrödinger′s tools with standard settings and Glide was used for docking and scoring. The 3D X-ray protein structures of FLT3 wildtype as a complex with a ligand were obtained from the PDB (code: 4RT7) and prepared using the Protein Preparation Wizard of the Schrödinger Maestro program. All water molecules were removed from the structure and it was selected as a template. The structures of inhibitors were drawn using Chemdraw, and their 3D conformation was generated using the Schrödinger LigPrep program with the OPLS 2005 force field. Molecular docking of compound into the structure of FLT-3 wildtype (PDB code: 4RT7) were carried out using Schrodinger Glide (Version 11.5).

2. Chemistry

2.1 General chemical methods

All chemicals were of reagent grade and were purchased from Aldrich (USA). Separation of the compounds by column chromatography was carried out with silica gel 60 (200−300 mesh ASTM, E. Merck, Germany). The quantity of silica gel used was 50−100 times the weight charged on the column. Thin layer chromatography (TLC) was run on the silica gel-coated aluminum sheets (silica gel 60 GF254, E. Merck, Germany) and visualized under ultraviolet (UV) light (254 nm). 1H NMR and ^{13}C NMR spectra were recorded on a Brucker model digital AVANCE III 400 MHz spectrometer at 25 ^{o}C using tetramethylsilane (TMS) as an internal standard. High-resolution MS (HR/MS) experiments were conducted with a Finnigan LTQ Orbitrap mass spectrometer (Thermo Fisher Scientific Inc, MA, USA) operated in positive-ion electrospray mode.

2.2. General syntheses of N-(2-amino-3-nitrophenyl)-3-(4-methylpiperazin-1-yl)-5-(trifluoromethyl)benzamide (2a)

A solution of 1,2,-diamino-3-nitrobenzene (100 mg, 0.653 mmol), triethylamine (0.18 ml, 1.306 mmol), and 3-(4-methylpiperazin-1-yl)-5-(trifluoromethyl)benzoyl chloride (240.3 mg, 0.784 mmol) in a mixture of CH_2Cl_2/acetonitrile (2/1, 4.90 ml) was stirred at room temperature for 2h. After this time, the reaction mixture was diluted with CH_2Cl_2 and washed with water. The two layers were separated, and the organic layer was dried over $MgSO_4$, filtered, and concentrated. Purification of column chromatography with MC/Methanol = 15 : 1 to afford compound 2a as a yellow solid (164.6 mg, 59.53%); ^{1}H NMR (400 MHz, DMSO) δ 10.00 (s, 1H), 8.00 (dd, J = 8.7, 1.5 Hz, 1H), 7.82 (s, 1H), 7.67 (s, 1H), 7.47 (dd, J = 7.4, 1.2 Hz, 1H), 7.37 (s, 1H), 7.22 (s, 2H), 6.70 (dd, J = 8.7, 7.5 Hz, 1H), 3.32 (d, J = 8.0 Hz, 5H), 2.49 – 2.44 (m, 5H), 2.24 (s, 3H); HRMS (ESI+) calculated for $C_{19}H_{20}F_3N_5O_3$ [M+H]+: 424.1518, found 424.4466.

N-(2-amino-3-nitrophenyl)-4-morpholino-3-(trifluoromethyl)benzamide (2b)

Purification of column chromatography with EA/Hex = 1 : 1 to afford compound 2b as a yellow solid in 74.1% yield; ^{1}H NMR (400 MHz, DMSO) δ 10.01 (s, 1H), 8.33 (d, *J* = 1.9 Hz, 1H), 8.28 (dd, *J* = 8.4, 1.8 Hz, 1H), 7.99 (dd, *J* = 8.8, 1.5 Hz, 1H), 7.66 (d, *J* = 8.5 Hz, 1H), 7.46 (dd, *J* = 7.4, 1.4 Hz, 1H), 7.23 (s, 2H), 6.69 (dd, *J* = 8.7, 7.4 Hz, 1H), 3.78 – 3.69 (m, 4H), 3.02 – 2.92 (m, 4H); HRMS (ESI+) calculated for $C_{18}H_{20}F_3N_5O_3$ [M+H]+: 411.1202, found 411.2744.

N-(2-amino-3-nitrophenyl)-3-(4-methyl-1H-imidazol-1-yl)-5 (trifluoromethyl) benzamide (2c)

Purification of column chromatography with MC/Methanol = 25 : 1 to afford compound 2c as a yellow solid in 68.0% yield; ^{1}H NMR (400 MHz, DMSO) δ 10.15 (s, 1H), 8.53 (s, 1H), 8.40 (d, *J* = 1.2 Hz, 1H), 8.27 (s, 1H), 8.22 (s, 1H), 8.02 (dd, *J* = 8.7, 1.5 Hz, 1H), 7.71 (s, 1H), 7.52 (dd, *J* = 7.4, 1.4 Hz, 1H), 7.31 (s, 2H), 6.72 (dd, *J* = 8.7, 7.5 Hz, 1H), 2.19 (d, *J* = 0.7 Hz, 3H); HRMS (ESI+) calculated for $C_{18}H_{14}F_3N_5O_3$ [M+H]+: 406.1049, found 406.2008.

2.3. General syntheses of 2-(3-(4-methylpiperazin-1-yl)-5-(trifluoromethyl)phenyl)-5-nitro-1H-benzo[d]imidazole (3a-3j)

4-Nitro-1,2-phenylenediamine (200 mg, 1.31mmol) and 3-(4-Methyl-piperazin-1-yl)-5-trifluoromethyl-benzoic acid (564.72mg, 1.96mmol) were taken in reaction vial and phosphorus oxychloride (1.9ml) was added slowly. The reaction mixture was allowed to stir under microwave irradiation (192 °C, 10min). After 10min, the reaction was allowed to cool and then poured slowly into an ice water mixture in an Erlenmeyer flask with vigorous stirring. Greenish yellow precipitate fell out which was then filtered and washed with copious amounts of water. The residue was then dried to obtain 529.41 mg of crude desired product. The reaction mixture diluted with ethyl acetate and washed with 1M NaOH. The organic layer dried over $MgSO_4$. Purification of column chromatography with MC/MeOH = 20 : 1 to afford compound ***3a*** as a yellow solid (334.8 mg, 63%); ^{1}H NMR (400 MHz, DMSO) δ 10.79 (s, 1H), 8.50 (s, 1H), 8.16 (s, 2H), 8.00 (s, 1H), 7.82 (s, 1H), 7.49 (s, 1H), 4.17-3.19(4H, m) 2.85 (s, 3H); HRMS (ESI+) calculated for $C_{19}H_{18}F_3N_5O_2$ [M+H]+: 406.1413, found 406.1557.

4-(3-(5-nitro-1H-benzo[d]imidazol-2-yl)-5-(trifluoromethyl)phenyl)morpholine (3b) : The title compound mixture was isolated as yellow solid in 49% yield; ^{1}H NMR (400 MHz, DMSO) δ 13.78 (d, *J* = 7.9 Hz, 1H), 8.57-8.41 (m, 3H), 8.16 (t, *J* = 10.8 Hz, 1H), 7.88-7.76 (m, 2H), , 3.80 – 3.72 (m, 4H), 3.05 – 2.96 (m, 4H); HRMS (ESI+) calculated for $C_{18}H_{15}F_3N_4O_3$ [M+H]+: 393.1096, found 393.0186.

4-(4-(5-nitro-1H-benzo[d]imidazol-2-yl)-2-(trifluoromethyl)phenyl)morpholine (3c) : The title compound mixture was isolated as yellow solid in 32.5% yield; ^{1}H NMR (400 MHz, DMSO) δ 13.78 (d, *J* = 7.9 Hz, 1H), 8.57-8.41 (m, 3H), 8.16 (t, *J* = 10.8 Hz, 1H), 7.88-7.76 (m, 2H), , 3.80 – 3.72 (m, 4H), 3.05 – 2.96 (m, 4H); HRMS (ESI+) calculated for

$C_{18}H_{15}F_3N_4O_3$ [M+H]+: 393.1096, found 393.0186.

2-(3-(4-methyl-1H-imidazol-1-yl)-5-(trifluoromethyl)phenyl)-5-nitro-1H-benzo[d]imidazole (3d) : The title compound mixture was isolated as yellow solid in 58.4% yield; ^{1}H NMR (400 MHz, DMSO) δ 13.86 (s, 1H), 8.62 (s, 1H), 8.52 (s, 1H), 8.41 (s, 1H), 8.39 (d, *J* = 1.1 Hz, 1H), 8.20 (s, 1H), 8.16 (dd, *J* = 8.9, 2.1 Hz, 1H), 7.83 (d, *J* = 8.9 Hz, 1H), 7.68 (s, 1H), 2.20 (s, 3H); HRMS (ESI+) calculated for $C_{18}H_{12}F_3N_5O_2$ [M+H]+: 388.0943, found 388.1040.

2-(3-(5-methyl-1H-imidazol-1-yl)-5-(trifluoromethyl)phenyl)-5-nitro-1H-benzo[d] imidazole (3e) : The title compound mixture was isolated as yellow solid in 54.1% yield; ^{1}H NMR (400 MHz, DMSO) δ 13.94 (s, 1H), 8.63 (s, 1H), 8.56 (s, 1H), 8.51 (s, 1H), 8.18 (dd, *J* = 8.9, 2.1 Hz, 1H), 8.13 (s, 1H), 8.00 (s, 1H), 7.86 (d, *J* = 8.7 Hz, 1H), 6.92 (s, 1H), 2.27 (d, *J* = 0.6 Hz, 3H); HRMS (ESI+) calculated for $C_{18}H_{15}F_3N_4O_3$ [M+H]+: 388.0943, found 388.2615.

2-(3-(2-methyl-1H-imidazol-1-yl)-5-(trifluoromethyl)phenyl)-5-nitro-1H-benzo [d]imidazole (3f) : The title compound mixture was isolated as yellow solid in 43.0% yield; ^{1}H NMR (400 MHz, DMSO) δ 13.95 (s, 1H), 8.62 (s, 1H), 8.52 (s, 1H), 8.19 (d, *J* = 8.8 Hz, 1H), 8.13 (s, 1H), 7.87 (s, 1H), 7.55 (s, 1H), 7.02 (s, 1H), 2.40 (s, 3H); HRMS (ESI+) calculated for $C_{18}H_{15}F_3N_4O_3$ [M+H]+: 388.0943, found 388.1040.

5-nitro-2-(1-phenyl-5-(trifluoromethyl)-1H-pyrazol-4-yl)-1H-benzo[d]imidazole (3g) : The title compound mixture was isolated as yellow solid in 42% yield; ^{1}H NMR (400 MHz, DMSO) δ 13.63 (s, 1H), 8.58- 8.43 (m, 2H), 8.22 – 8.09 (s, 1H), 7.92 – 7.73 (s, 1H), 7.65 – 7.59 (m, 5H); HRMS (ESI+) calculated for $C_{17}H_{10}F_3N_5O_2$ [M+H]+: 374.0787, found 374.0847.

2.4. General syntheses of 2-(3-(4-methylpiperazin-1-yl)-5-(trifluoromethyl)phenyl)-4-nitro-1H-benzo[d]imidazole (3h) : A solution of **2h** (100 mg, 0.236mmol) and concd aq HCl (25.19 ul) in acetic acid (0.708 ml) was submitted to microwave irradiation at 150 °C, for 30 min. The reaction mixture was cooled to room temperature, and the product precipitated upon cooling. The precipitate was filtered, washed with acetic acid and ether, and then dried to provide **3h** as HCl salt (95.5 mg, 91.6%); ^{1}H NMR (400 MHz, DMSO) δ 13.40 (s, 1H), 10.73 (s, 1H), 8.29 – 8.14 (m, 2H), 8.05 (d, *J* = 15.8 Hz, 1H), 7.48 (t, *J* = 8.0 Hz, 2H), 3.53 (s, 4H), 3.25 (d, *J* = 22.4 Hz, 4H), 2.84 (s, 3H); HRMS (ESI+) calculated for $C_{19}H_{18}F_3N_5O_2$ [M+H]+: 406.1413, found 406.1378.

4-(4-(4-nitro-1H-benzo[d]imidazol-2-yl)-2-(trifluoromethyl)phenyl)morpholine (3i) : The title compound mixture was isolated as yellow solid in 73.6% yield; ^{1}H NMR (400 MHz, DMSO) δ 8.73 (d, *J* = 2.1 Hz, 1H), 8.64 (dd, *J* = 8.5, 2.1 Hz, 1H), 8.17 (ddd, *J* = 8.0, 5.8, 0.9 Hz, 2H), 7.72 (d, *J* = 8.6 Hz, 1H), 7.49 (t, *J* = 8.1 Hz, 1H), 3.81 – 3.72 (m, 4H), 3.08 – 2.95 (m, 4H); HRMS (ESI+) calculated for $C_{18}H_{15}F_3N_4O_3$ [M+H]+: 393.1096, found 393.5010.

2-(3-(4-methyl-1H-imidazol-1-yl)-5-(trifluoromethyl)phenyl)-4-nitro-1H-benzo [d]imidazole (3j) : The title compound mixture was isolated as yellow solid in 91.8% yield; ^{1}H

NMR (400 MHz, DMSO) δ 13.49 (s, 1H), 8.90 (s, 1H), 8.66 (s, 1H), 8.44 (s, 1H), 8.22 (d, *J* = 6.9 Hz, 3H), 7.74 (s, 1H), 7.51 (t, *J* = 8.1 Hz, 1H), 2.22 (d, *J* = 0.7 Hz, 3H); HRMS (ESI+) calculated for $C_{18}H_{12}F_3N_5O_2$ [M+H]+: 388.0943, found 388.1437.

2.5. General syntheses of 2-(3-(4-methylpiperazin-1-yl)-5-(trifluoromethyl)phenyl)-1H-benzo[d]imidazol-5-amine (4a) : A suspension of 3a (69 mg, 0.17 mmol) and 7 mg of Pd/C (10%) in 3ml of methanol was stirred for 2hrs under H_2. After filtering through celite, the solution was concentrated under reduced pressure to give 0.17 mmol of 4a. Title crude compound was used as a starting material for next step without further purification; HRMS (ESI+) calculated for $C_{19}H_{20}F_3N_5$ [M+H]+: 376.1324, found 376.1324.

2-(3-morpholino-5-(trifluoromethyl)phenyl)-1H-benzo[d]imidazol-5-amine (4b) : The title compound mixture was isolated as pure solid in 73.7% yield; ^{1}H NMR (400 MHz, DMSO) δ 12.44 (s, 1H), 7.86 (s, 1H), 7.80 (s, 1H), 7.31 (d, *J* = 6.8 Hz, 1H), 7.22 (s, 1H), 6.66 (s, 1H), 6.55 (d, *J* = 7.6 Hz, 1H), 5.04 (s, 2H), 3.85 – 3.72 (m, 4H), 3.33 – 3.21 (m, 4H); HRMS (ESI+) calculated for $C_{18}H_{17}F_3N_4O$ [M+H]+: 363.1354, found 363.2218.

2-(4-morpholino-3-(trifluoromethyl)phenyl)-1H-benzo[d]imidazol-5-amine (4c) : The title compound mixture was isolated as pure solid in 80% yield; ^{1}H NMR (400 MHz, DMSO) δ 12.43 (s, 1H), 8.36 (s, 1H), 8.30 (d, *J* = 8.3 Hz, 1H), 7.67 (d, *J* = 8.5 Hz, 1H), 7.30 (d, *J* = 6.9 Hz, 1H), 6.67 (s, 1H), 6.54 (d, *J* = 8.0 Hz, 1H), 5.00 (s, 2H), 3.79 – 3.66 (m, 4H), 2.99 – 2.86 (m, 4H); HRMS (ESI+) calculated for $C_{18}H_{17}F_3N_4O$ [M+H]+: 363.1354, found 363.2794.

2-(3-(4-methyl-1H-imidazol-1-yl)-5-(trifluoromethyl)phenyl)-1H-benzo[d] imidazole-5-amine (4d) : 3d (60 mg, 0.16 mmol) and $SnCl_22H_2O$ (174.78 mg, 0.77 mmol) in EtOH (0.77 mL) was stirred at 80°C. Stirring was continued for 2h and the clear solution was cooled to room temperature. Solvent was removed in vacuo. The pH was made slightly basic (pH 7–8) by addition of saturated aqueous sodium bicarbonate before being extracted with ethyl acetate. The organic phase is thoroughly washes with brine, dried over magnesium sulfate to produce 0.155 mmol of 4d as a yellow solid; ^{1}H NMR (400 MHz, DMSO) δ 8.65 (s, 1H), 8.57 (s, 1H), 8.36 (s, 1H), 8.09 (s, 1H), 7.78 (s, 1H), 7.42 (d, *J* = 8.2 Hz, 1H), 6.87 (s, 1H), 6.71 (d, *J* = 7.8 Hz, 1H); HRMS (ESI+) calculated for $C_{18}H_{14}F_3N_5$ [M+H]+: 358.1201, found 358.1118.

2-(3-(5-methyl-1H-imidazol-1-yl)-5-(trifluoromethyl)phenyl)-1H-benzo[d] imidazol-5-amine (4e) : The title compound mixture was isolated as pure solid in 99.2% yield; ^{1}H NMR (400 MHz, DMSO) δ 12.72 (d, 1H), 8.51 (d, 1H), 8.37 (d, 1H), 8.06 – 7.88 (m, 2H), 7.33 (dd, 1H), 6.88 (d, 1H), 6.68 (t, 1H), 6.59 (dd, 1H), 5.16 (s, 2H), 2.27 (d, *J* = 0.9 Hz, 3H); HRMS (ESI+) calculated for $C_{18}H_{14}F_3N_5$ [M+H]+: 358.1201, found 358.1118.

2-(3-(2-methyl-1H-imidazol-1-yl)-5-(trifluoromethyl)phenyl)-1H-benzo[d] imidazol-5-amine (4f) : The title compound mixture was isolated as pure solid in 100% yield. Crude compound was used as a starting material for next step without further purification; HRMS (ESI+) calculated for $C_{18}H_{14}F_3N_5$ [M+H]+: 358.1201, found 358.1433.

2-(1-phenyl-5-(trifluoromethyl)-1H-pyrazol-4-yl)-1H-benzo[d]imidazol-5-amine (4g): The title compound mixture was isolated as pure solid in 76.9% yield.; ^{1}H NMR (400 MHz, DMSO) δ 12.42-12.27 (s, 1H), 8.30 (s, 1H), 7.65 – 7.54 (m, 5H), 7.34-7.25 (d, J = 8.3 Hz, 1H), 6.82-6.68 (s, 1H), 6.56 (d, J = 8.0 Hz, 1H), 5.07 (s, 2H) ; HRMS (ESI+) calculated for $C_{17}H_{12}F_3N_5$ [M+H]+: 344.1045, found 344.1867.

2-(3-(4-methylpiperazin-1-yl)-5-(trifluoromethyl)phenyl)-1H-benzo[d] imidazole-4-amine (4h) : The title compound mixture was isolated as pure solid in 97.2% yield; ^{1}H NMR (400 MHz, DMSO) δ 12.99-12.77 (s, 1H), 7.92 (d, J = 25.1 Hz, 2H), 7.62 (dd, J = 8.1, 5.7 Hz, 1H), 7.27 (s, 1H), 6.92 (t, J = 7.7 Hz, 1H), 6.71 (d, J = 7.5 Hz, 1H), 6.36 (d, J = 7.3 Hz, 1H), 5.34 (s, 2H), 3.45 (s, 4H), 2.78 (s, 4H), 2.44 (s, 3H); HRMS (ESI+) calculated for $C_{19}H_{20}F_3N_5$ [M+H]+: 376.1671, found 376.1684.

2-(4-morpholino-3-(trifluoromethyl)phenyl)-1H-benzo[d]imidazol-4-amine (4i): The title compound mixture was isolated as pure solid in 80.9% yield. Crude compound was used as a starting material for next step without further purification; HRMS (ESI+) calculated for $C_{18}H_{17}F_3N_4O$ [M+H]+: 363.1354, found 363.4681.

2-(3-(4-methyl-1H-imidazol-1-yl)-5-(trifluoromethyl)phenyl)-1H-benzo[d] imidazol-4-amine (4j) : 3j (50 mg, 0.13 mmol) and Fe (36.02 mg, 0.645 mmol) in EtOH/AcOH/H_2O (2:2:1, 1.29 mL) was stirred at 60°C. Stirring was continued for 1h and the clear solution was cooled to room temperature. Solvent was removed in vacuo. Then after filtering through celite, the solution was concentrated under reduced pressure. The pH was made slightly basic (pH 7–8) by addition of 1M aqueous sodium hydroxide before being extracted with ethyl acetate. The organic phase is thoroughly washes with brine, dried over magnesium sulfate to produce 4j as a solid.(42 mg, 91.11%);^{1}H NMR (400 MHz, DMSO) δ 12.85-12.68 (s, 1H), 8.58-8.54 (s, 1H), 8.41 (s, 1H), 8.37-8.31 (s, 1H), 8.12-8.09 (s, 1H), 7.71-7.66 (s, 1H), 6.97 (t, J = 7.8 Hz, 1H), 6.75 (d, J = 7.9 Hz, 1H), 6.52 -6.39 (m, J = 7.6 Hz, 1H), 5.43-5.20 (s, 1H), 2.21 (s, 3H) ; HRMS (ESI+) calculated for $C_{18}H_{14}F_3N_5$ [M+H]+: 358.1201, found 358.4258.

2.6. 5-methylisoxazole-4-carbonyl chloride: The 5-methylisoxazole-4-carboxylic acid (1 g, 7.86 mmol) in $SOCl_2$ (3 mL) was heated at 50°C until compound acid disappeared in TLC. After reaction termination, the mixture was cooled to ambient temperature and solvent was evaporated under reduced pressure. 5-methylisoxazole-4-carbonyl chloride (6) as a crude yellow oil (96%) was used for the next step without further purification; ^{1}H NMR (400 MHz, DMSO) δ8.77 (1H, s), 2.64 (3H, s).

2.7. General syntheses of 5-methyl-N-(2-(3-(4-methylpiperazin-1-yl)-5-(trifluoromethyl)phenyl)-1H-benzo[d]imidazol-5-yl)isoxazole-4-carboxamide (5a~6c)

The mixture of 5-methylisoxazole-4-carbonyl chloride (32.45 mg, 0.26 mmol) and 4a (63.85 mg, 0.17 mmol) in THF (1.7 ml) was heated at 65°C until compound 3a disappeared in TLC. After completion of the reaction, the mixture was cooled to ambient temperature and solvent was removed in vacuo. The reaction mixture diluted with ethyl acetate and washed with saturated aqueous sodiumbicarbonate. The organic layer dried over $MgSO_4$. The concentrated

crude product was purified by preparative TLC with MC/methanol (10:1) to afford compound 5a as a pure solid (4a) as a yellow solid (2.8%); ^{1}H NMR (400 MHz, MeOD) δ 8.77 (s, 1H), 8.04 (s, 1H), 7.83 (s, 1H), 7.73 (s, 1H), 7.50 (s, 1H), 7.33 (s, 1H), 7.22 (s, 1H), 3.37 – 3.31 (m, 4H), 2.65 (m, 4H), 2.62 (s, 3H), 2.33 (s, 3H); ^{13}C NMR (101 MHz, DMSO) δ 171.11 (s), 166.88 (s), 161.33, 155.62, 150.92, 141.10, 138.86, 132.69, 126.00, 125.72, 123.29, 116.39, 116.37, 116.35, 114.33, 114.29, 113.57, 113.56, 77.64, 52.85, 45.83, 27.56; HRMS (ESI+) calculated for $C_{24}H_{23}F_3N_6O_2$ [M+H]+: 485.1835, found 485.1334.

5-methyl-N-(2-(3-morpholino-5-(trifluoromethyl)phenyl)-1H-benzo[d] imidazole-5-yl)isoxazole-4-carboxamide (5b) : The title compound mixture was isolated as pure solid in 66.8% yield; ^{1}H NMR (400 MHz, DMSO) δ 13.05 (s, 1H), 10.12 (s, 1H), 9.10 (s, 1H), 8.11 (s, 1H), 7.95 (s, 1H), 7.88 (s, 1H), 7.62 (d, *J* = 8.6 Hz, 1H), 7.45 (d, *J* = 8.3 Hz, 1H), 7.31 (s, 1H), 3.87 – 3.75 (m, 4H), 3.34 – 3.27 (m, 4H), 2.71 (s, 3H); ^{13}C NMR (101 MHz, DMSO) δ 172.52, 159.04, 151.81, 150.83, 150.50, 150.49, 149.10, 131.71, 130.87, 130.55, 125.56, 122.85, 115.29, 112.60, 112.56, 112.16, 111.76, 65.87, 47.68, 12.12; HRMS (ESI+) calculated for $C_{24}H_{23}F_3N_6O_2$ [M+H]+: 472.1518, found 472.3238.

5-methyl-N-(2-(4-morpholino-3-(trifluoromethyl)phenyl)-1H-benzo[d] imidazol-5-yl)isoxazole-4-carboxamide (5c) : The title compound mixture was isolated as pure solid in 86.3% yield; ^{1}H NMR (400 MHz, DMSO) δ 13.05 (s, 1H), 10.11 (s, 1H), 9.10 (s, 1H), 8.46 (d, *J* = 1.9 Hz, 1H), 8.40 (dd, *J* = 8.4, 1.8 Hz, 1H), 8.10 (s, 1H), 7.71 (d, *J* = 8.5 Hz, 1H), 7.60 (d, *J* = 8.6 Hz, 1H), 7.43 (d, *J* = 8.1 Hz, 1H), 3.78 – 3.68 (m, 4H), 3.00 – 2.89 (m, 4H), 2.71 (s, 3H); ^{13}C NMR (101 MHz, DMSO) δ 172.52, 159.01, 152.75, 152.74, 150.05, 149.08, 131.08, 126.53, 125.62, 125.34, 125.29, 125.11, 125.05, 125.00, 124.86, 122.57, 112.15, 109.63, 66.48, 53.19, 12.11; HRMS (ESI+) calculated for $C_{23}H_{20}F_3N_5O_3$ [M+H]+: 472.1518, found 472.3238.

5-methyl-N-(2-(3-(4-methyl-1H-imidazol-1-yl)-5-(trifluoromethyl)phenyl)-1H-benzo[d]imidazol-5-yl)isoxazole-4-carboxamide (5d) : The title compound mixture was isolated as pure solid in 6.8% yield; ^{1}H NMR (400 MHz, DMSO) δ 13.25 (s, 1H), 10.16 (s, 1H), 9.12 (s, 1H), 8.64 (s, 1H), 8.51 (s, 1H), 8.41 (s, 1H), 8.16-8.15 (m, 2H), 7.76 (s, 1H), 7.72 – 7.58 (m, 1H), 7.50 (s, 1H), 2.71 (s, 3H), 2.21 (s, 3H); ^{13}C NMR (101 MHz, DMSO) δ 173.08, 163.41, 159.60, 149.62, 144.22, 138.51, 135.59, 135.55, 133.32, 132.44, 132.10, 131.77, 125.35, 121.70, 121.14, 121.08, 118.04, 118.02, 115.28, 115.23, 112.64, 13.51, 12.64; HRMS (ESI+) calculated for $C_{23}H_{17}F_3N_6O_2$ [M+H]+: 467.1365, found 467.1456.

5-methyl-N-(2-(3-(5-methyl-1H-imidazol-1-yl)-5-(trifluoromethyl)phenyl)-1H-benzo[d]imidazol-5-yl)isoxazole-4-carboxamide (5e) : The title compound mixture was isolated as pure solid in 4.1% yield; ^{1}H NMR (400 MHz, DMSO) δ 13.25 (s, 1H), 10.15 (s, 1H), 9.11 (s, 1H), 8.57 (s, 1H), 8.44 (s, 1H), 8.17 (s, 1H), 8.03 (s, 1H), 7.65 (dd, *J* = 41.3, 6.3 Hz, 1H), 7.49 (dd, *J* = 36.0, 9.0 Hz, 1H), 6.95 (s, 1H), 2.70 (s, 3H), 2.27 (s, 3H); ^{13}C NMR (101 MHz, DMSO) δ 173.11, 163.82, 159.62, 149.58, 142.05, 139.66, 138.93, 135.58, 135.23, 133.12, 128.14, 127.09, 124.02, 123.39, 122.73, 122.55, 119.62, 116.37, 115.57, 112.62, 112.21, 12.62, 10.03; HRMS (ESI+) calculated for $C_{23}H_{17}F_3N_6O_2$ [M+H]+: 467.1365, found 467.1456.

5-methyl-N-(2-(3-(2-methyl-1H-imidazol-1-yl)-5-(trifluoromethyl)phenyl)-1H-benzo[d]imidazol-5-yl)isoxazole-4-carboxamide (5f) : The title compound mixture was

isolated as pure solid in 8.04% yield; ^{1}H NMR (400 MHz, DMSO) δ 13.24 (s, 1H), 10.14 (s, 1H), 9.11 (s, 1H), 8.56 (s, 1H), 8.45 (s, 1H), 8.18 – 8.11 (s, 1H), 8.02 (s, 1H), 7.72 – 7.44 (m, 3H), 7.01 (s, 1H), 2.71 (s, 3H), 2.40 (s, 3H); ^{13}C NMR (101 MHz, DMSO) δ 173.09, 163.14, 159.60, 149.60, 139.17, 138.87, 135.65, 133.15, 132.18, 132.14, 131.81, 131.49, 127.17, 126.90, 125.26, 123.43, 122.66, 122.54, 121.88, 119.70, 112.63, 13.77, 12.63; HRMS (ESI+) calculated for $C_{23}H_{17}F_3N_6O_2$ [M+H]+: 467.1365, found 467.1456.

5-methyl-N-(2-(1-phenyl-5-(trifluoromethyl)-1H-pyrazol-4-yl)-1H-benzo[d] imidazol-5-yl)isoxazole-4-carboxamide (5g) : The title compound mixture was isolated as pure solid in 65.6% yield; ^{1}H NMR (400 MHz, DMSO) δ 12.90-12.86 (s, 1H), 10.14-10.08 (s, 1H), 9.12 (s, 1H), 8.38 (s, 1H), 8.17-8.09 (s, 1H), 7.68-7.54 (m, 6H), 7.49-7.40 (dd, *J* = 8.7, 1.8 Hz, 1H), 2.71 (s, 3H); ^{13}C NMR (101 MHz, DMSO) δ 173.04, 159.54, 149.58, 143.52, 141.20, 139.50, 134.21, 130.46, 129.87, 129.25, 128.87, 126.52, 121.42, 118.74, 116.82, 116.81, 116.77, 116.46, 112.66, 12.61; HRMS (ESI+) calculated for $C_{22}H_{15}F_3N_6O_2$ [M+H]+: 453.1209, found 453.0955.

5-methyl-N-(2-(3-(4-methylpiperazin-1-yl)-5-(trifluoromethyl)phenyl)-1H-benzo[d]imidazol-4-yl)isoxazole-4-carboxamide (6a) : The title compound mixture was isolated as pure solid in 29.8% yield; ^{1}H NMR (400 MHz, DMSO) δ 13.21-12.53 (s, 1H), 10.23-10.11 (s, 1H), 9.27-9.19 (s, 1H), 8.03 (s, 1H), 7.97-7.93 (s , 1H), 7.74-7.60 (d, *J* = 7.9 Hz, 1H), 7.42 – 7.38 (m, 1H), 7.33 (d, *J* = 7.5 Hz, 1H), 7.25 (m, *J* = 16.2, 8.0 Hz, 1H), 3.38 (d, *J* = 4.5 Hz, 4H), 2.74 (d, *J* = 6.6 Hz, 3H), 2.55 (m, 4H), 2.29 (s, 3H); ^{13}C NMR (101 MHz, DMSO) δ 173.08, 159.86, 154.55, 151.97, 150.15, 149.86, 137.04, 135.98, 132.02, 126.07, 123.58, 123.36, 116.29, 115.69, 113.24, 112.64, 112.49, 108.42, 54.54, 47.56, 45.73, 12.67; HRMS (ESI+) calculated for $C_{24}H_{23}F_3N_6O_2$ [M+H]+: 485.1835, found 485.2003.

5-methyl-N-(2-(4-morpholino-3-(trifluoromethyl)phenyl)-1H-benzo[d] imidazol-4-yl)isoxazole-4-carboxamide (6b) : The title compound mixture was isolated as pure solid in 86.3% yield; ^{1}H NMR (400 MHz, DMSO) δ 13.21-12.51 (s, 1H), 10.12 (s, 1H), 9.21 (s, 1H), 8.53 (s, 1H), 8.48 (dd, *J* = 8.5, 1.8 Hz, 1H), 7.73 (d, *J* = 8.5 Hz,1H), 7.61-7.30 (m, 2H), 7.23 (t, *J* = 7.9 Hz, 1H), 3.78 – 3.70 (m, 4H), 3.00 – 2.93 (m, 4H), 2.73 (s, 3H); ^{13}C NMR (101 MHz, DMSO) δ 173.21, 159.81, 153.44, 149.86, 132.00, 126.65, 126.01, 125.99, 125.97, 125.95, 125.82, 125.69, 125.22, 123.09, 123.06, 123.04, 123.02, 112.52, 66.97, 53.68, 12.68; HRMS (ESI+) calculated for $C_{23}H_{20}F_3N_5O_3$ [M+H]+: 472.1518, found 472.2495.

5-methyl-N-(2-(3-(4-methyl-1H-imidazol-1-yl)-5-(trifluoromethyl)phenyl)-1H-benzo[d]imidazol-4-yl)isoxazole-4-carboxamide (6c) : The title compound mixture was isolated as pure solid in 35.0% yield; ^{1}H NMR (400 MHz, DMSO) δ 13.33-12.62 (s, 1H), 10.31-10.08 (s, 1H), 9.26- 9.17 (s, 1H), 8.67 (s, 1H), 8.48 (d, *J* = 6.2 Hz, 1H), 8.39 (s, 1H), 8.15 (s, 1H), 7.78 (d, *J* = 7.7 Hz)-7.65 (d, *J* = 15.3 Hz, 2H), 7.42 (d, *J* = 8.0 Hz)-7.28 (t, *J* = 7.7 Hz, 2H), 2.73 (s, 3H), 2.21 (s, 3H); ^{13}C NMR (101 MHz, DMSO) δ 173.43, 160.96, 159.88, 149.83, 140.10, 137.79, 137.76, 135.48, 135.44, 133.29, 133.26, 132.32, 132.29, 131.82, 131.76, 125.76, 125.25, 118.34, 117.05, 112.49, 112.46, 12.73, 12.70; HRMS (ESI+) calculated for $C_{23}H_{17}F_3N_6O_2$ [M+H]+: 467.1365, found 467.1763.

3. Evaluation of IC_{50} and Selected Kinase Profiling

We used Reaction Biology Corp. *Kinase HotSpot*^{SM} service (www.reactionbiology.com) for IC_{50} determination of all compounds and kinase profile. Assay protocol: In a final reaction volume of 25 μL, Peptide substrate, [EAIYAAPFAKKK], 5 μM, ATP 10 μM , FLT3(h) (5-10 mU) is incubated with 25 mM Tris pH 7.5, 0.02 mM EGTA, 0.66 mg/mL myelin basic protein, 10 mM Mg Acetate and [γ-33P-ATP] (specific activity approx. 500 cpm/pmol, concentration as required). The reaction is initiated by the addition of the Mg-ATP mix. After incubation for 40 minutes at room temperature, the reaction is stopped by the addition of 5 μL of a 3% phosphoric acid solution. 10 μL of the reaction is then spotted onto a P30 filtermat and washed three times for 5 minutes in 75 mM phosphoric acid and once in methanol prior to drying and scintillation counting.

4. ^{1}H NMR Spectra of Final products

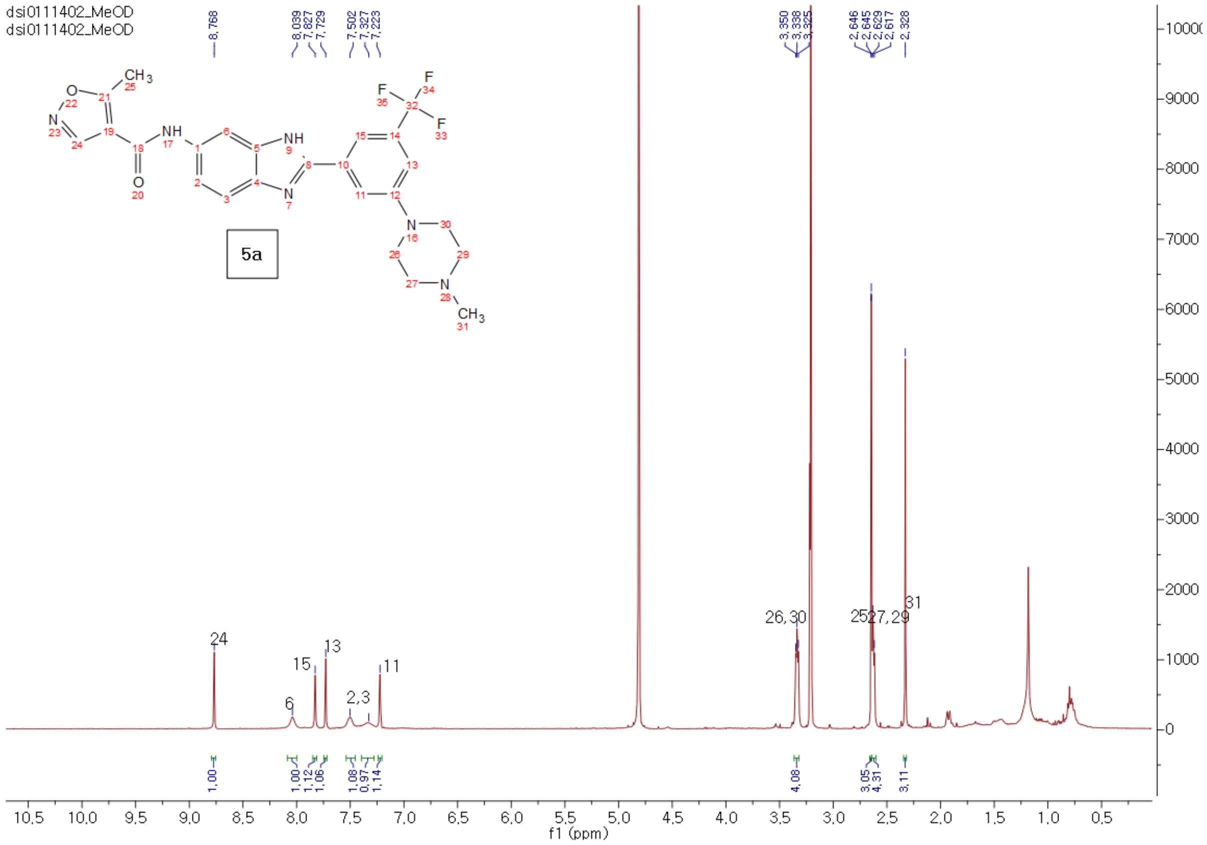

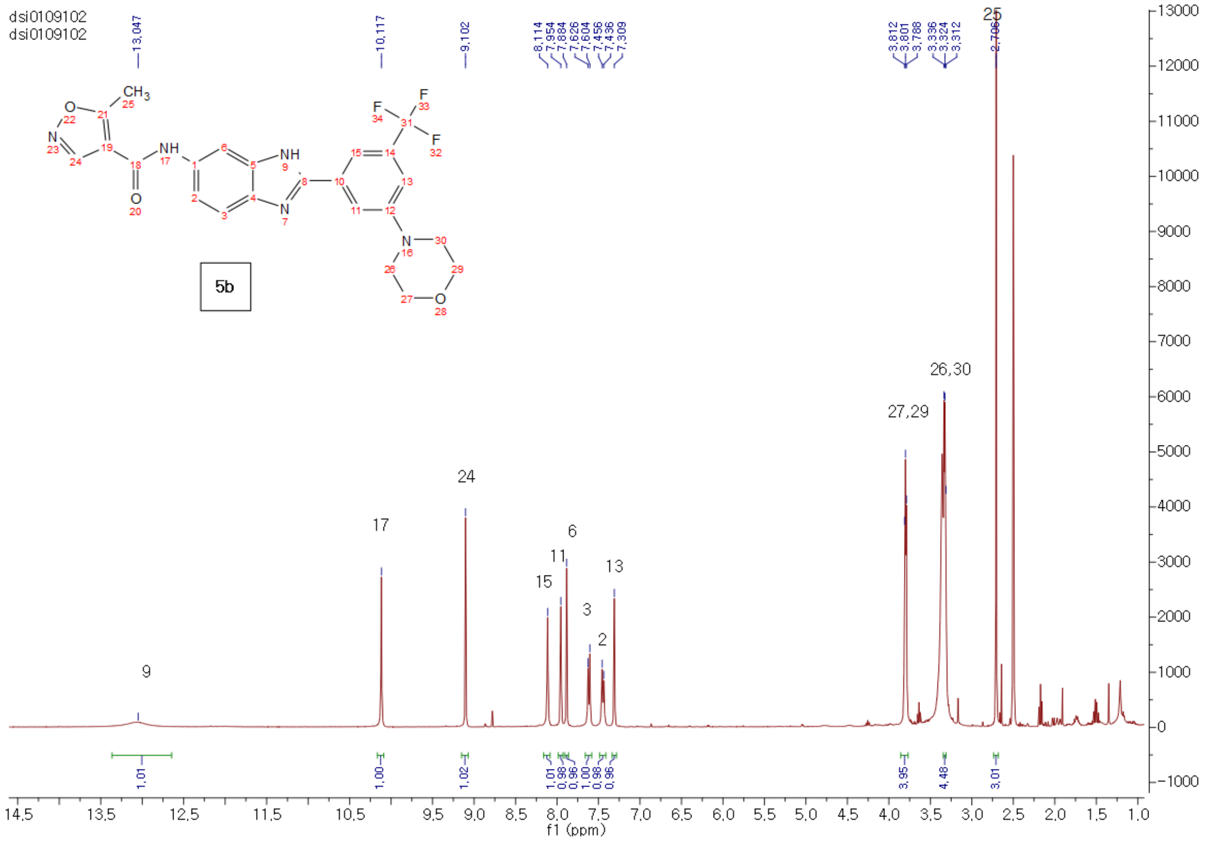
dsi0109102
dsi0109102
13.047
10.117
9.102
8.114 7.954 7.884 7.626 7.604 7.456 7.436 7.309
3.812 3.801 3.788 3.336 3.324 3.312
2.706
5b
9
17
24
15
11
6
3
2
13
27,29
26,30
25
1.01
1.00
1.02
1.01 0.98 0.96 1.00 0.98 0.96
3.95
4.48
3.01
f1 (ppm)

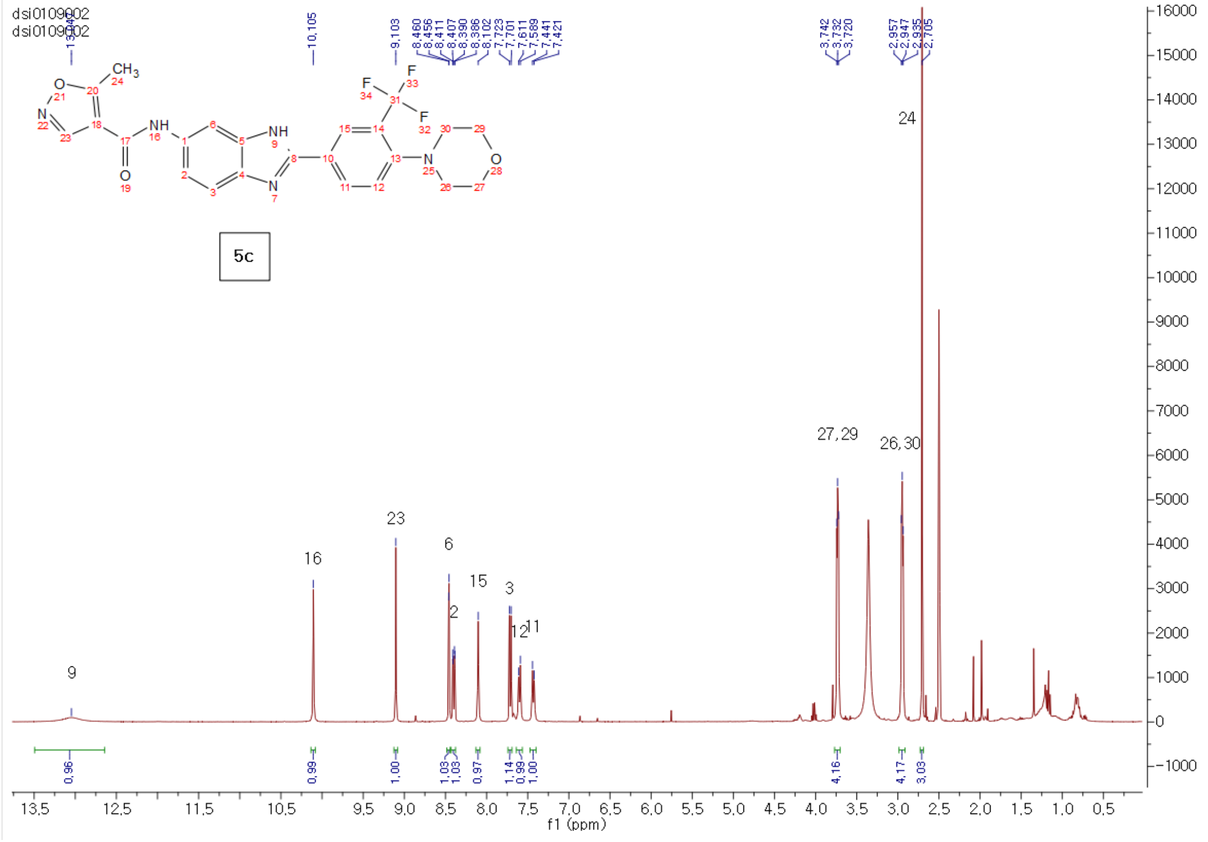
10.105
9.103
8.460 8.456 8.411 8.407 8.390 8.386 8.102 7.723 7.701 7.611 7.589 7.441 7.421
3.742 3.732 3.720
2.957 2.947 2.935 2.705
5c
9
16
23
6
2
15
3
12
11
27,29
26,30
24
0.96
0.99
1.00
1.03 1.03
0.97
1.14 0.99 1.00
4.16
4.17
3.03
f1 (ppm)

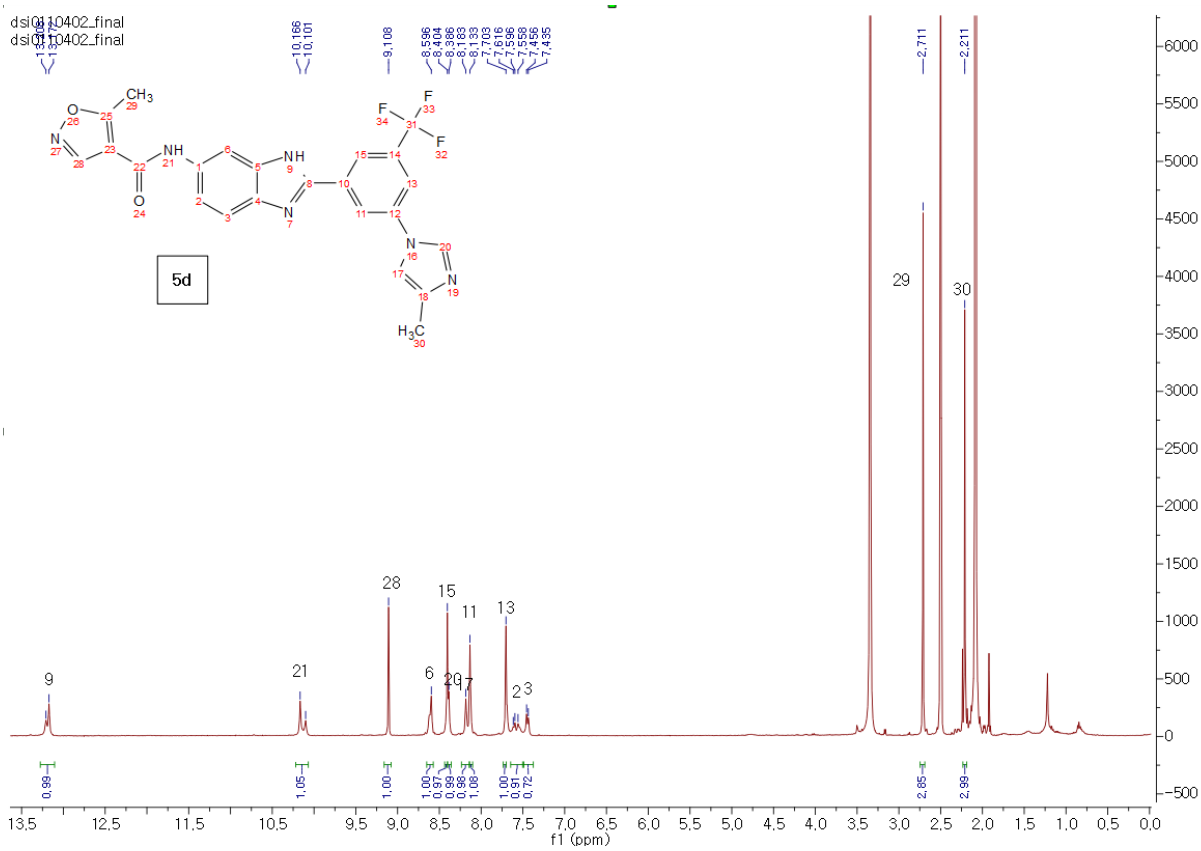
dsi0110402_final
5d
CH3
NH
F
f1 (ppm)

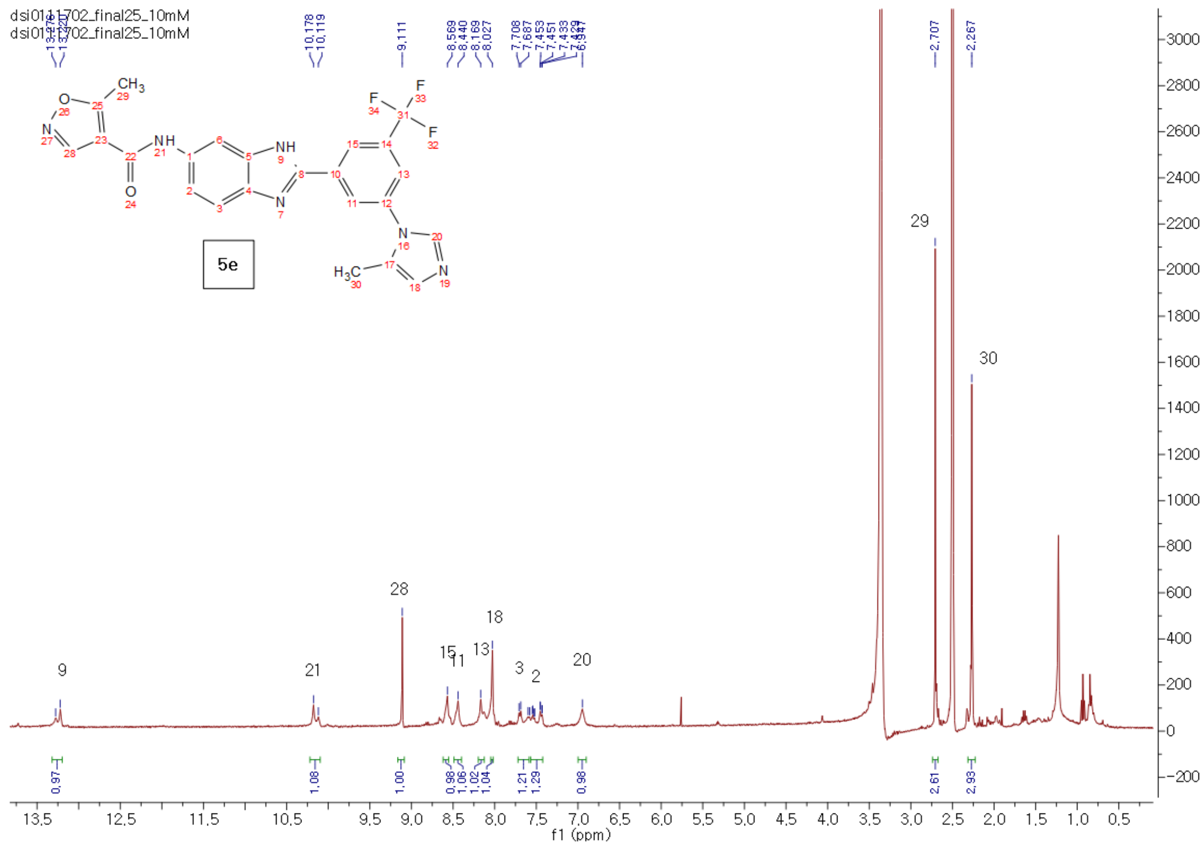
dsi0111702_final25_10mM
5e
CH3
H3C
NH
F
f1 (ppm)

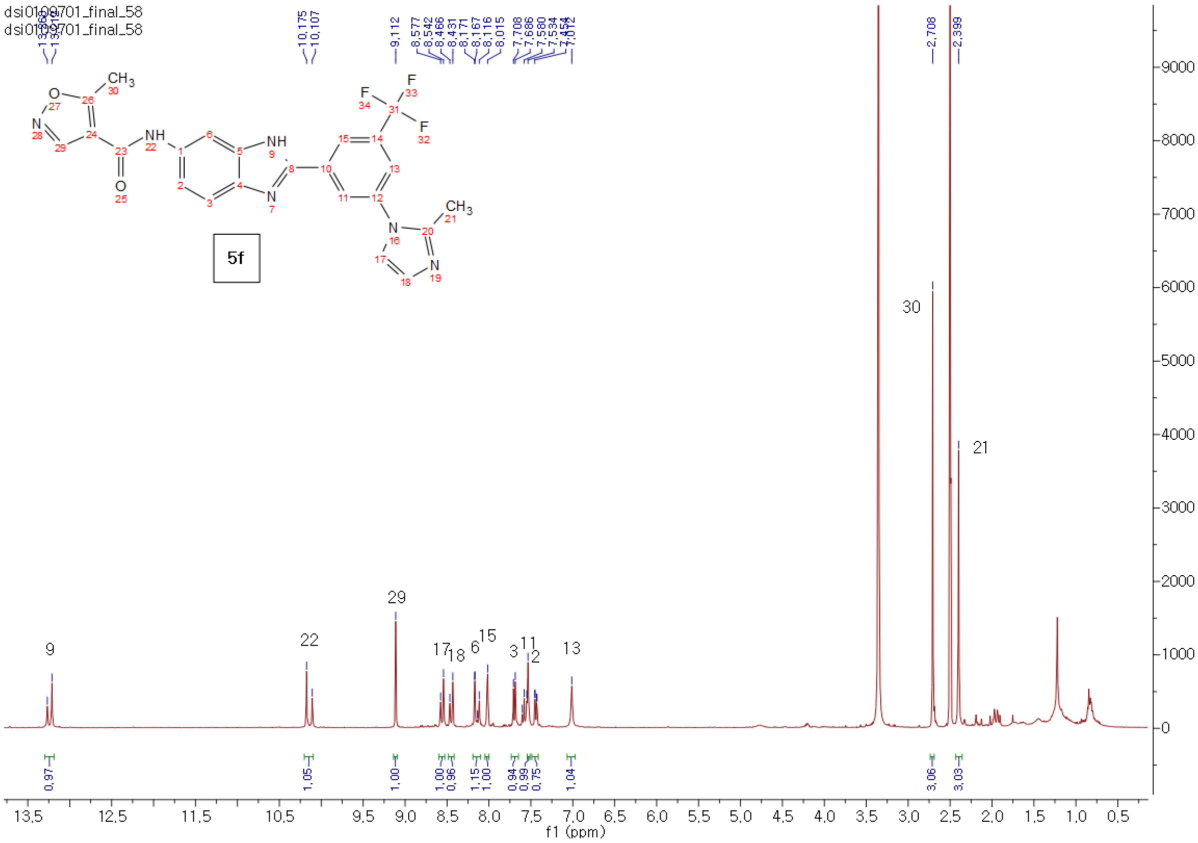
dsi0109701_final_58
5f
f1 (ppm)

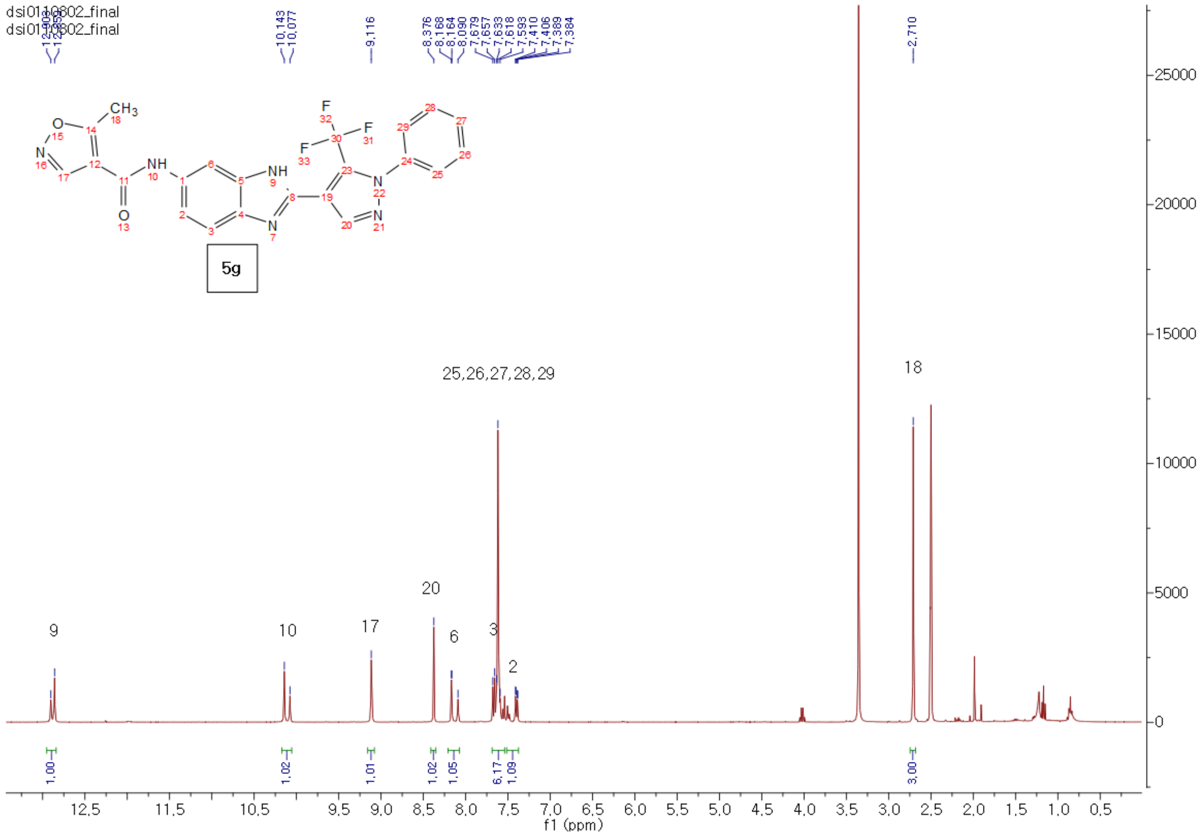
dsi0110802_final
5g
25,26,27,28,29
f1 (ppm)

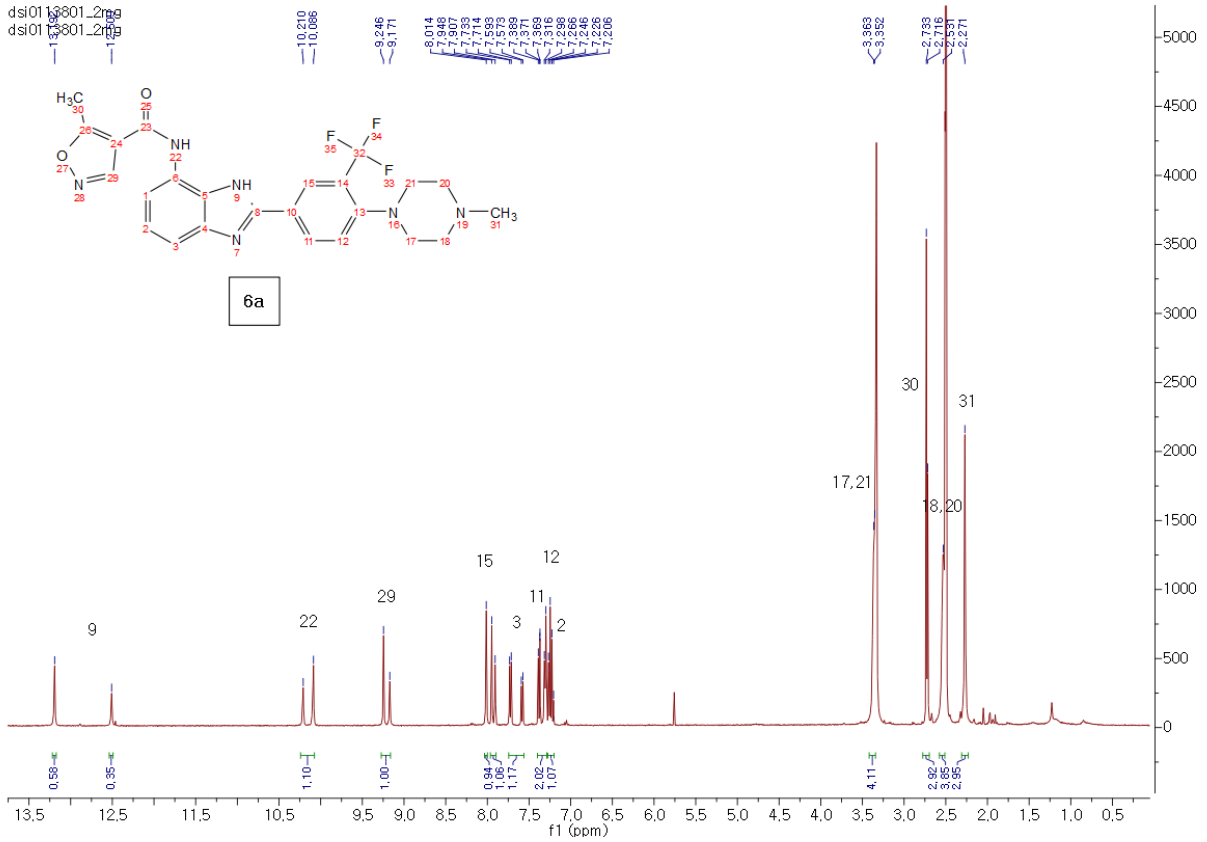
dsi0113801_2mg
dsi0113801_2mg
6a
9
22
29
15
3
11
12
2
17,21
30
18,20
31
f1 (ppm)

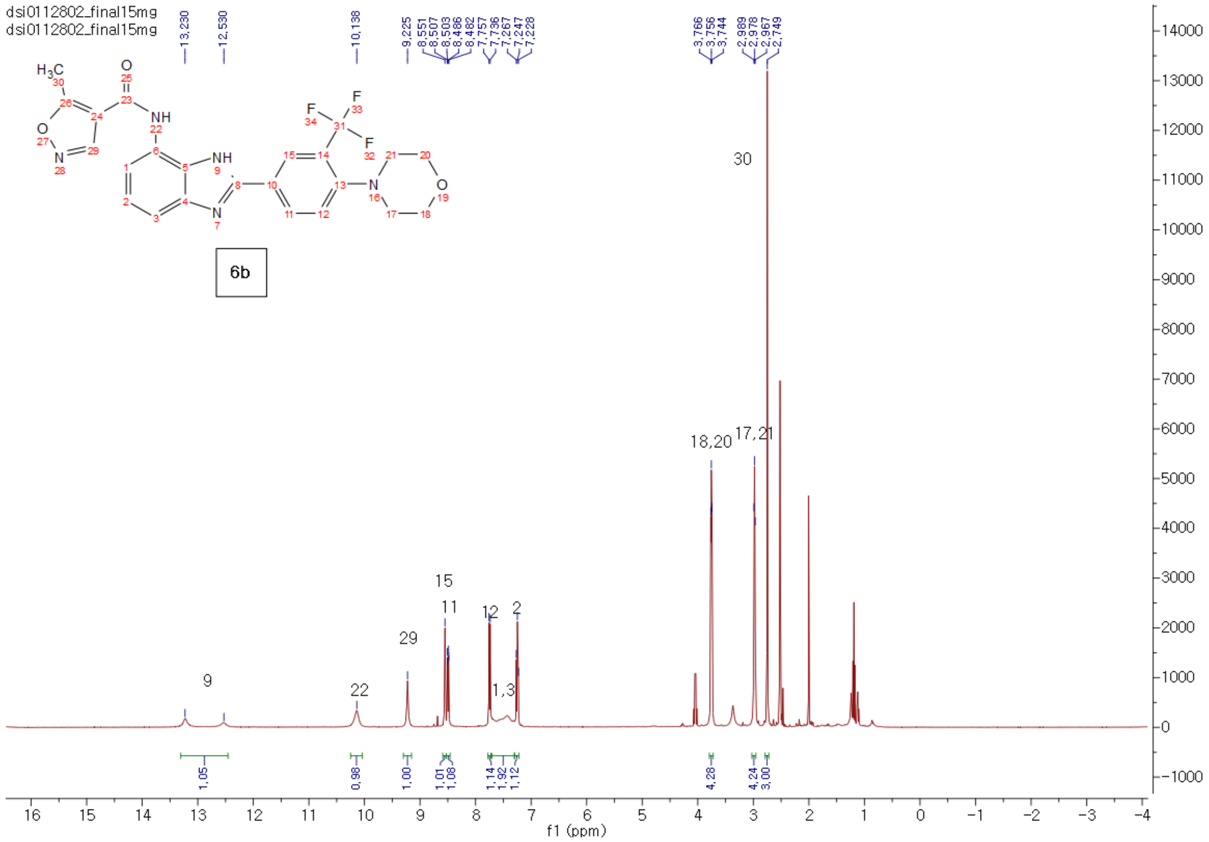
dsi0112802_final15mg
dsi0112802_final15mg
6b
9
22
29
15
11
1,3
12
2
18,20
17,21
30
f1 (ppm)

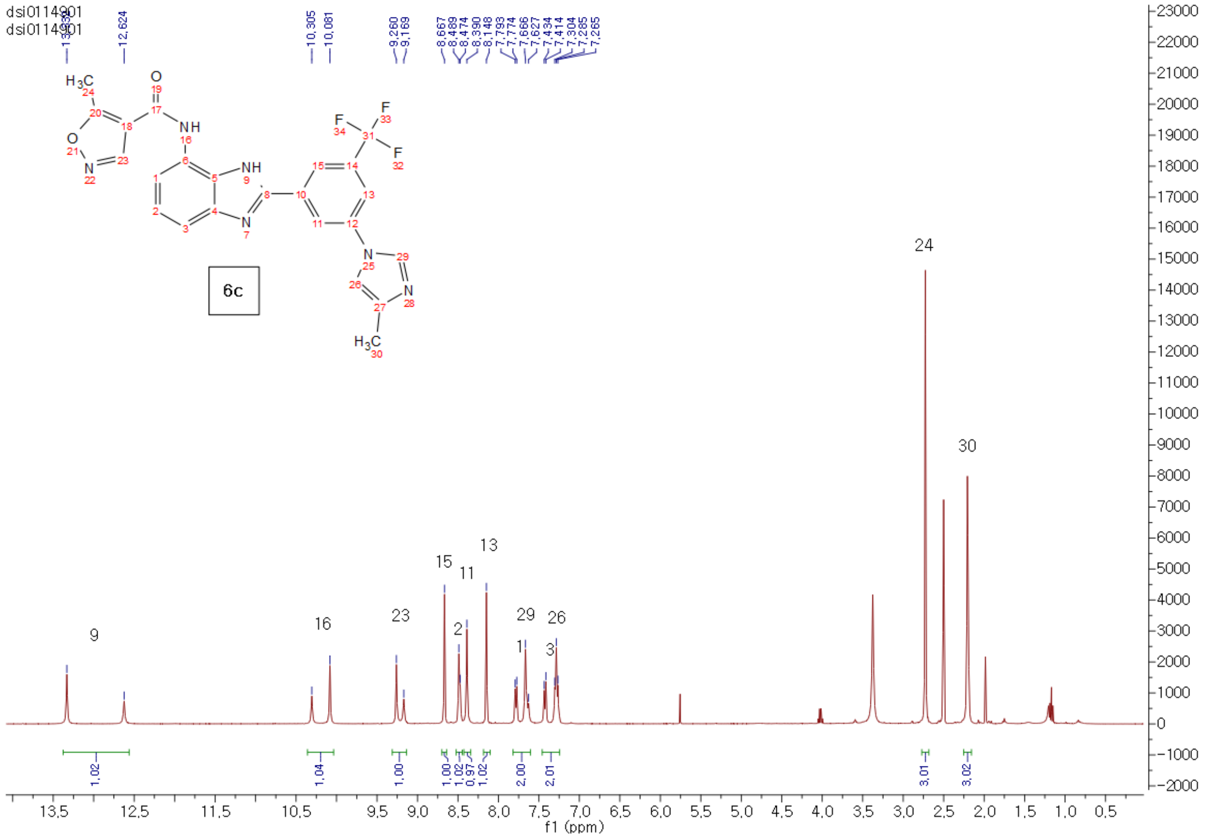
dsi0114901
dsi0114901
12,624
10,305
10,081
9,260
9,169
8,667
8,489
8,474
8,390
8,148
7,793
7,774
7,666
7,627
7,434
7,414
7,304
7,285
7,265
6c
f1 (ppm)
